# A metabolic epidemic? Prevalence and sex-based disparities of metabolic alterations in the peruvian population using multiple diagnostic criteria

**DOI:** 10.1007/s40200-025-01622-8

**Published:** 2025-04-29

**Authors:** Víctor Juan Vera-Ponce, Luisa Erika Milagros Vásquez-Romero, Fiorella E. Zuzunaga-Montoya, Joan A. Loayza-Castro, Jhonatan Roberto Astucuri Hidalgo, Carmen Inés Gutierrez De Carrillo

**Affiliations:** 1 Instituto de Investigación de Enfermedades Tropicales, Universidad Nacional Toribio Rodríguez de Mendoza de Amazonas (UNTRM), Amazonas, Perú; 2 Facultad de Medicina (FAMED), Universidad Nacional Toribio Rodríguez de Mendoza de Amazonas (UNTRM), Amazonas, Perú; 3 Universidad Continental, Lima, Perú; 4 Universidad Tecnológica del Perú, Lima, Perú

**Keywords:** Metabolism, Body mass index, Waist circumference, Metabolism

## Abstract

**Introduction:**

Metabolic alterations constitute a growing challenge for global public health, with significant implications for cardiovascular morbidity and mortality. Early identification of these alterations, even from the presence of a single component, is crucial for effectively preventing and managing chronic diseases.

**Objective:**

To determine the prevalence of metabolic states based on one or more alterations in the Peruvian population and to evaluate possible sex disparities.

**Methods:**

An analytical cross-sectional study used data from two Peruvian national databases: Surveillance of Nutritional Indicators by Life Stages (VIANEV) and PERU MIGRANT. Data from 885 adults from VIANEV and 986 participants from PERU MIGRANT with complete information for all study variables were analyzed. Graphs were generated to illustrate metabolic states according to different combinations of diagnostic criteria. Bar charts were created to visualize the individual prevalences of each state. Ordinal logistic regression was employed to examine sex disparities and the outcome.

**Results:**

The prevalence of metabolic alterations (at least one alteration) ranged from 87.04% to 87.55%, depending on the criteria used. Significant discrepancies were found in the prevalences of hyperglycemia and abdominal obesity according to the different diagnostic criteria applied. The ordinal logistic regression analysis showed that men had a lower probability of presenting metabolic alterations compared to women, regardless of the diagnostic method used.

**Conclusions:**

This study reveals a high prevalence of metabolic alterations in the Peruvian population, with notable variations depending on the diagnostic criteria employed. The observed discrepancies underscore the need to re-evaluate these criteria for the Peruvian population. The identified disparities between sexes suggest the importance of developing differentiated prevention and management strategies.

## Introduction

Metabolic alterations represent a growing challenge for global public health, with significant implications for cardiovascular morbidity and mortality. Traditionally, research and clinical focus has centered on metabolic syndrome, defined by the presence of three or more metabolic alterations, or the unhealthy metabolic state, characterized by two or more alterations [1]. However, growing evidence suggests that even a single metabolic alteration can have significant long-term health impacts [2], underscoring the importance of adopting a more comprehensive approach to metabolic risk assessment.

The prevalence of metabolic alterations varies significantly across different populations and regions worldwide. In Latin America, previous studies have reported prevalence rates of metabolic syndrome ranging from 18.8% to 43.3% [3, 4], with considerable heterogeneity between and within countries. Peru, as a middle-income country experiencing rapid epidemiological transition, faces a substantial burden of metabolic disorders, yet comprehensive data on the full spectrum of metabolic alterations in the Peruvian population remains limited.

Furthermore, recent research has revealed significant differences in the manifestation and impact of metabolic alterations according to sex [5]. These disparities may stem from biological differences in fat distribution, hormonal influences on metabolism, and social determinants of health that affect dietary patterns and physical activity levels differentially between men and women. Understanding these sex-based disparities is crucial for developing more effective and personalized prevention and management strategies.

An additional complexity in assessing metabolic alterations lies in the varying diagnostic criteria proposed by different international organizations. The Adult Treatment Panel III (ATP III) and the International Diabetes Federation (IDF) offer different cut-off points for abdominal obesity [6, 7], while the American Diabetes Association (ADA) and the World Health Organization (WHO) propose distinct thresholds for defining hyperglycemia [8, 9]. These differences in criteria can significantly impact reported prevalence rates and, consequently, public health policies and clinical guidelines.

In this context, the present study aims to determine the prevalence of metabolic states based on one or more alterations in the Peruvian population and to evaluate possible sex disparities. By examining the prevalence of metabolic alterations using different diagnostic criteria and exploring differences between men and women, this study seeks to provide essential evidence to inform more effective and targeted public health interventions in Peru and potentially other Latin American countries.

## Methods

### Study design

This research employed an analytical cross-sectional design to assess the prevalence of metabolic alterations and evaluate potential sex disparities in the Peruvian population. We conducted a secondary analysis of data from two nationally representative studies with complementary strengths: the Surveillance of Nutritional Indicators by Life Stages (VIANEV) [10] study, which offers recent nationwide data with strong urban–rural representation, and the PERU MIGRANT study [11], which provides detailed information on different migration contexts. This dual-database approach allowed us to examine metabolic alterations across diverse demographic and geographic contexts within Peru.

### Populations and sample

We analyzed data from two complementary Peruvian studies: VIANEV (Surveillance of Nutritional Indicators by Life Stages) and PERU MIGRANT. These studies were selected because they offer comprehensive metabolic health data and together provide a more complete representation of the Peruvian population across different geographic, socioeconomic, and migratory contexts.

The VIANEV study (2017) [10] was designed to evaluate the nutritional status and associated risk factors in the Peruvian population across different life stages. It employed multi-stage probabilistic sampling at the national level, ensuring representation of both urban and rural areas across Peru’s diverse geographical regions. Initially, VIANEV included 1,211 adults aged 18–59 years.

The PERU MIGRANT study (2008) [11] was specifically designed to examine the impact of rural–urban migration on cardiovascular risk factors. It used random probabilistic sampling, focusing on three distinct populations: rural residents from the Ayacucho region, migrants from Ayacucho to Lima, and urban residents born in Lima. This study initially recruited 989 participants aged ≥ 30 years, providing valuable insights into how migration patterns influence metabolic health.

For our analysis, we included only participants with complete data for all anthropometric measurements and biochemical analyses required to assess the various metabolic components. After applying this criterion, our final analytical sample consisted of 885 participants from VIANEV and 986 participants from PERU MIGRANT. This inclusive combining both databases while maintaining their analytical independence, allowed us to obtain more robust and generalizable findings about metabolic alterations in the Peruvian population.

### Variables

The following variables were evaluated using globally standardized cut-off points:

1. Abdominal obesity (AO) according to Adult Treatment Panel III criteria (AO-ATP) [7]: waist circumference (WC) ≥ 102 cm in men or ≥ 88 cm in women
2. AO according to International Diabetes Federation criteria (AO-IDF) [6]: WC ≥ 94 cm in men or ≥ 80 cm in women
3. Hyperglycemia according to the American Diabetes Association (Hyperglycemia-ADA) [9]: fasting glucose ≥ 100 mg/dL
4. Hyperglycemia according to the World Health Organization (Hyperglycemia-WHO) [8]: fasting glucose ≥ 110 mg/dL
5. Obesity/overweight [12]: if body mass index (BMI) was ≥ 25 kg/m.^2^
6. Hypertriglyceridemia [7]: if triglycerides ≥ 150 mg/dL
7. Hypercholesterolemia [7]: if total cholesterol ≥ 200 mg/dL
8. Low HDL [7]: if HDL < 40 mg/dL in men and < 50 mg/dL in women
9. Elevated blood pressure [7]: if systolic blood pressure (SBP) ≥ 130 mmHg or diastolic blood pressure (DBP) ≥ 85 mmHg [13]

Four distinct versions of the Metabolic State variable were created from these criteria, each based on a different combination of criteria for AO and hyperglycemia. These variables were coded on an ordinal scale from 0 to 7, where 0 indicates the absence of any alteration of the metabolic state components and 7 indicates the presence of all components. The four combinations were:

- AO-ATP + Hyperglycemia-ADA: Uses the more conservative ATP III criteria for abdominal obesity (WC ≥ 102 cm in men, ≥ 88 cm in women) combined with the ADA criteria for hyperglycemia (fasting glucose ≥ 100 mg/dL)
- AO-IDF + Hyperglycemia-ADA: Uses the more sensitive IDF criteria for abdominal obesity (WC ≥ 94 cm in men, ≥ 80 cm in women) combined with the ADA criteria for hyperglycemia (fasting glucose ≥ 100 mg/dL)
- AO-ATP + Hyperglycemia-WHO: Uses ATP III criteria for abdominal obesity combined with WHO criteria for hyperglycemia (fasting glucose ≥ 110 mg/dL)
- AO-IDF + Hyperglycemia-WHO: Uses IDF criteria for abdominal obesity combined with WHO criteria for hyperglycemia

For each combination, a Metabolic State variable was created on an ordinal scale from 0 to 7, where 0 indicates the absence of any metabolic state components and 7 indicates the presence of all components (abdominal obesity, hyperglycemia, obesity/overweight, hypertriglyceridemia, hypercholesterolemia, low HDL, and elevated blood pressure).

The primary independent variable was sex, which was classified as male and female. Additionally, several covariables were analyzed in this study. Age was measured in years as a continuous variable. Educational level was categorized as none/primary, secondary, and higher education based on self-reported highest level of education completed. Area of residence was classified as urban versus rural according to the official definitions of the National Institute of Statistics and Informatics of Peru. Socioeconomic status was measured using the wealth index methodology, a composite measure based on household assets and housing characteristics, and categorized as non-poor versus poor. Physical activity was assessed using the International Physical Activity Questionnaire (IPAQ) and categorized as low, medium, and high according to standardized IPAQ scoring protocols. Smoking status was classified as current versus non-current smoker based on self-reported tobacco use in the last 30 days. Alcohol consumption was evaluated differently in each study due to methodological differences—in PERU MIGRANT as heavy drinker versus non-heavy drinker (based on consuming ≥ 6 alcoholic drinks on the same occasion at least monthly), and in VIANEV as excessive versus non-excessive consumption (based on consuming ≥ 5 standard drinks for men or ≥ 4 for women on a single occasion at least once in the past month).

## Procedures

For anthropometric measurements, in the VIANEV study, weight was measured with a digital scale (capacity 200 kg, precision 100 g) and height with a wooden stadiometer (precision 1 mm). In the PERU MIGRANT study, weight was recorded with a SECA 940 electronic scale (precision of 0.05 kg) and height with a stadiometer, with an accuracy of 0.1 cm. For WC, VIANEV used a retractable abdominal measuring tape (precision 0.1 cm), while PERU MIGRANT was measured in triplicate at the midpoint between the lower rib and the iliac crest.

For blood pressure measurement, VIANEV used an Omron automatic digital sphygmomanometer, taking two measurements and averaging them; in this case, the dominant arm was identified for blood pressure measurement, and if there was a difference of 20 mmHg in systolic pressure or ten mmHg in diastolic pressure between the first two measurements, a third measurement was taken, recording those that did not present such differences. Additionally, the timing of measurements was specified. PERU MIGRANT measured blood pressure using appropriate cuffs for arm circumference in a seated position, with the right arm supported at chest level. Three measurements were taken with a minimum interval of 5 min between them, using an Omron oscillometric device previously validated for the adult population. The average of the last two SBP and DBP measurements was taken for analysis.

Both VIANEV and PERU MIGRANT performed laboratory analyses on venous samples taken in the morning after a minimum 8-h fast by trained personnel. Fasting glucose was measured in plasma, serum, and whole blood. Additionally, VIANEV used previously calibrated portable glucometers and specified that the automated enzymatic-colorimetric method of coupled reactions at the endpoint was used to determine cholesterol and triglycerides. HDL and LDL determination was performed using the automated direct enzymatic colorimetric method.

### Statistical analysis

Statistical analysis was performed using R version 4.1.2 (R Foundation for Statistical Computing, Vienna, Austria) for descriptive and inferential analyses, while graphical representations were generated with Python version 3.9.7 using Matplotlib 3.5.1 and Seaborn 0.11.2 libraries.

A descriptive table of the study population characteristics was elaborated for the descriptive analysis. Age was presented as mean and standard deviation, while other categorical variables were presented as frequencies and percentages. Subsequently, four graphs were generated to illustrate metabolic states according to different combinations of criteria. Additionally, bar graphs were created to visualize the prevalences of each metabolic syndrome component separately.

To examine sex disparities in metabolic state, a bivariate analysis of each type of metabolic state was first presented. Additionally, ordinal logistic regression was used. This model was chosen due to the ordinal nature of the dependent variable (metabolic state), which was categorized from 0 to 7 according to the number of components present. No additional adjustments were made to the model due to the structure of the study variables. It was determined that most potential adjustment variables acted as mediators or colliders in the relationship between sex and metabolic state rather than actual confounding variables. This decision was based on the consideration that sex, as a biological variable, is not a result of other variables in the model but instead influences them.

### Ethical considerations

Both the PERU MIGRANT and VIANEV databases are freely accessible without restrictions. Therefore, the researchers did not consider it necessary to go through a research ethics committee. However, it is essential to emphasize that both databases were coded to avoid exposing names or any subject identification. Additionally, international standards of research ethics were complied with throughout the research process.

## Results

### Demographic characteristics – PERU MIGRANT/VIANEV

Comparing the PERU MIGRANT (*n* = 986) and VIANEV (*n* = 885) studies reveals both similarities and striking differences in Peruvian population characteristics. While both studies had similar gender distributions (approximately 53–56% female) and smoking rates (11–13% overall), they showed significant differences in several key areas. The PERU MIGRANT study reported a higher proportion of poor participants (35% versus 19% in VIANEV) and dramatically different residential patterns (80% urban in PERU MIGRANT versus 67% rural in VIANEV). Educational patterns revealed an interesting contrast: although PERU MIGRANT had more participants with secondary/higher education overall (52% versus 39%), this masked a substantial gender gap (64% of men versus 40% of women) that was less pronounced in VIANEV. Physical activity levels also differed considerably, with VIANEV participants reporting much higher rates of low physical activity (66% versus 26% in PERU MIGRANT), suggesting either genuine lifestyle differences between populations or methodological variations in measurement (Table 1).

**Table 1:** Demographic characteristics of the PERU MIGRANT/VIANEV studies

	PERU MIGRANT		VIANEV	
Characteristic	Female	Male	Female	Male
Age (mean ± DS)	47.78 ± 11.97	48.03 ± 11.94	38.80 ± 11.49	37.98 ± 12.34
Educational Level				
No Level/Primary	311 (59.69)	166 (35.85)	301 (61.55)	234 (59.69)
Secondary/Superior	210 (40.31)	297 (64.15)	188 (38.45)	158 (40.31)
Wealth index				
Poor	200 (38.39)	146 (31.40)	98 (19.92)	66 (16.79)
No poor	321 (61.61)	319 (68.60)	394 (80.08)	327 (83.21)
Area of residence				
Rural	105 (20.15)	95 (20.43)	341 (69.31)	257 (65.39)
Urban	416 (79.85)	370 (79.57)	151 (30.69)	136 (34.61)
Smoker Current				
No	502 (96.35)	374 (80.43)	458 (93.09)	305 (78.61)
Yes	19 (3.65)	91 (19.57)	34 (6.91)	83 (21.39)
Alcohol consumption 1				
Never or Non-excessive	—	—	485 (98.58)	379 (96.44)
Excessive	—	—	7 (1.42)	14 (3.56)
Alcohol consumption 2				
Light drinker	508 (97.50)	386 (83.01)	—	—
Heavy drinker	13 (2.50)	79 (16.99)	—	—
Physical Activity (MET score)				
Low	137 (26.50)	117 (25.38)	322 (65.45)	265 (67.43)
Moderate	180 (34.82)	105 (22.78)	100 (20.33)	74 (18.83)
High	200 (38.68)	239 (51.84)	70 (14.23)	54 (13.74)

*n* (%)

### Consistent patterns of metabolic alterations in peruvian populations – PERU MIGRANT/VIANEV

**Fig. 1:**
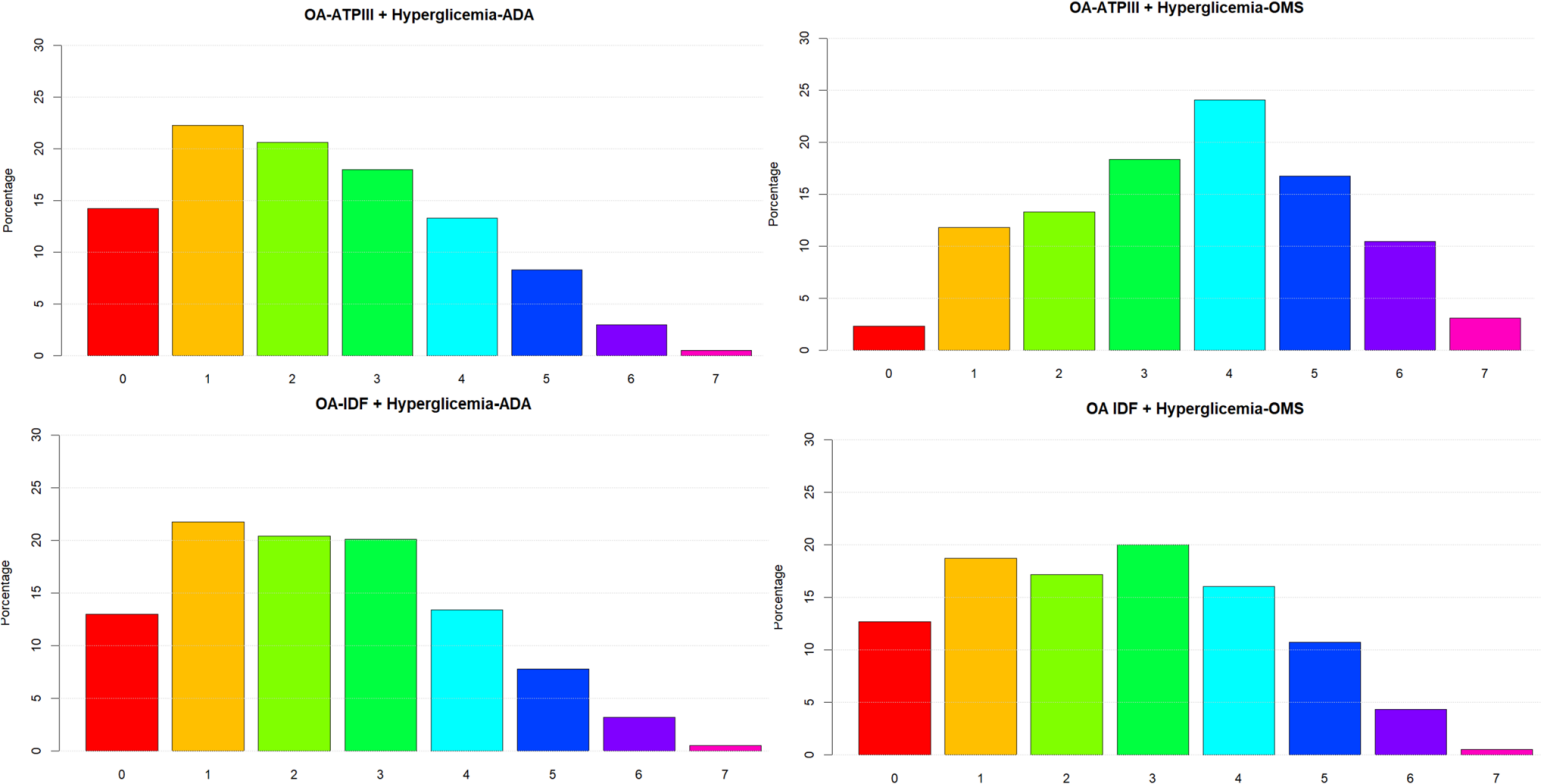
Distribution of metabolic alterations according to different diagnostic criteria in the population of the PERU MIGRANT study

**Fig. 2:**
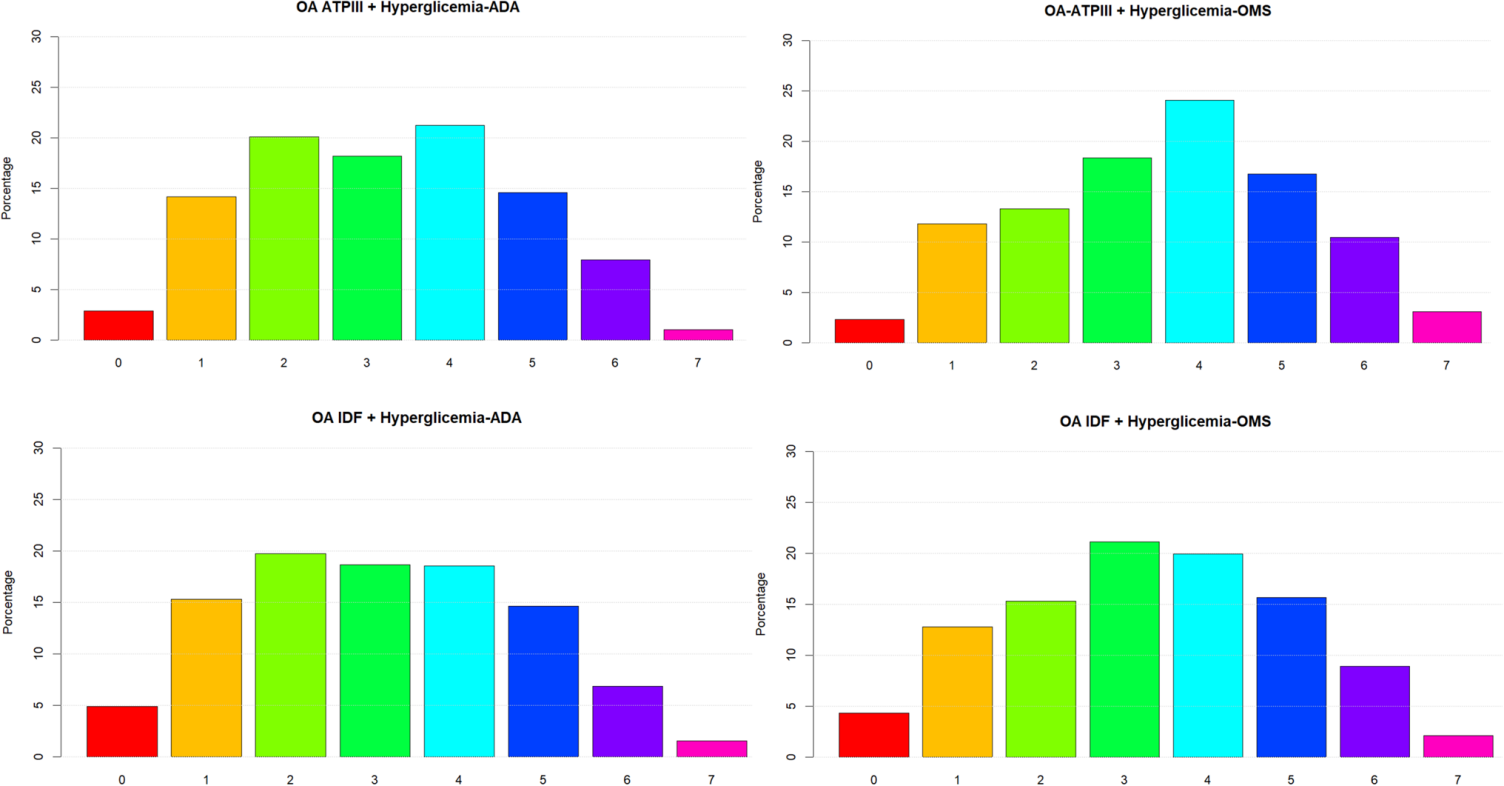
Distribution of metabolic alterations according to different diagnostic criteria in the VIANEV study population

Analysis and comparison of results from the PERU MIGRANT and VIANEV studies, as shown in Figs. 1 and 2, reveal notably similar patterns in the distribution of metabolic alterations, regardless of the diagnostic criteria used. Both studies evidence a significant prevalence of multiple metabolic alterations. The prevalence of metabolic alterations (with at least one alteration) ranged from 87.04% to 87.55%, depending on the criteria used.

Notably, the AO-IDF criteria with the ADA hyperglycemia definition consistently result in a higher prevalence of multiple alterations, with a peak at four alterations in both studies. This finding underscores the importance of diagnostic criteria selection in assessing population metabolic health.

Another striking aspect is the low proportion of individuals without metabolic alterations across all criteria combinations in PERU MIGRANT and VIANEV. This raises questions about the adequacy of current cut-off points for the Peruvian population or about the country’s general state of metabolic health.

Both studies exhibit a similar severity gradient, characterized by a gradual decrease in the proportion of individuals as the number of alterations increases. This consistent pattern reinforces the validity of the findings and suggests a distribution of metabolic risk that could be characteristic of the Peruvian population.

### Prevalence of metabolic disorders in the study populations

**Fig. 3:**
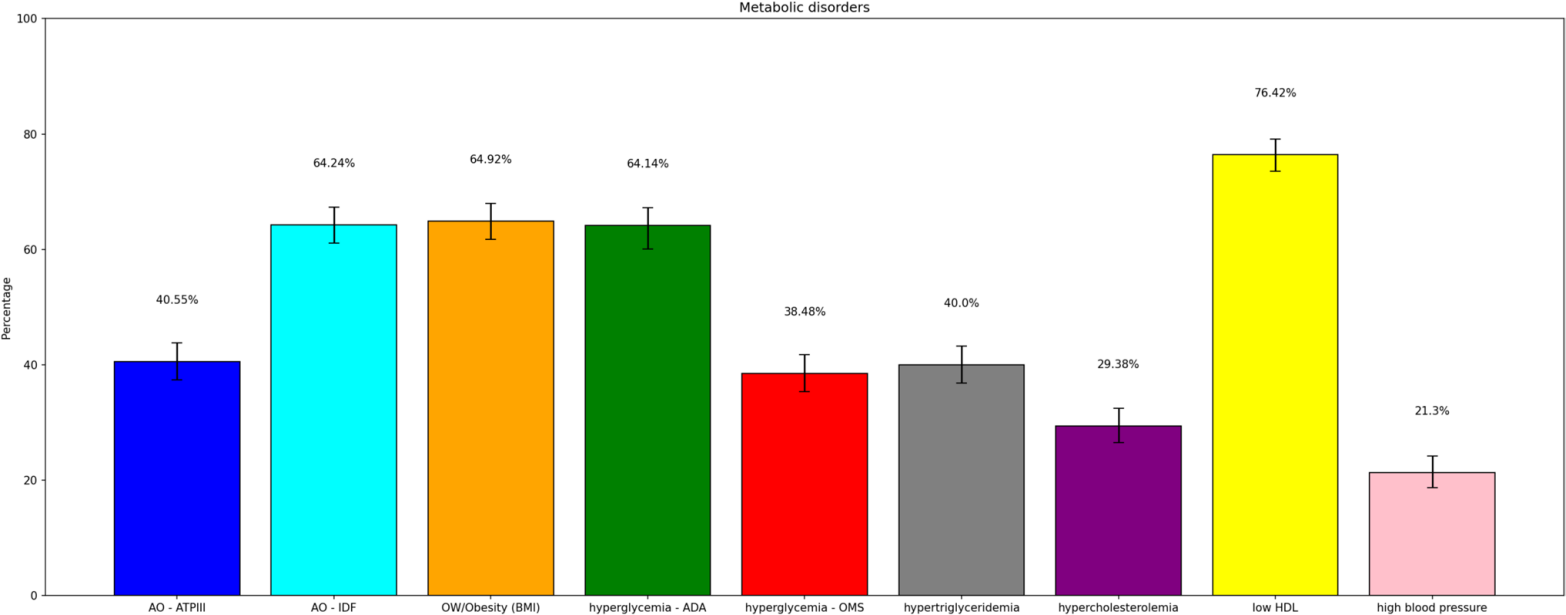
Prevalences of each component of the metabolic state – VIANEV

**Fig. 4:**
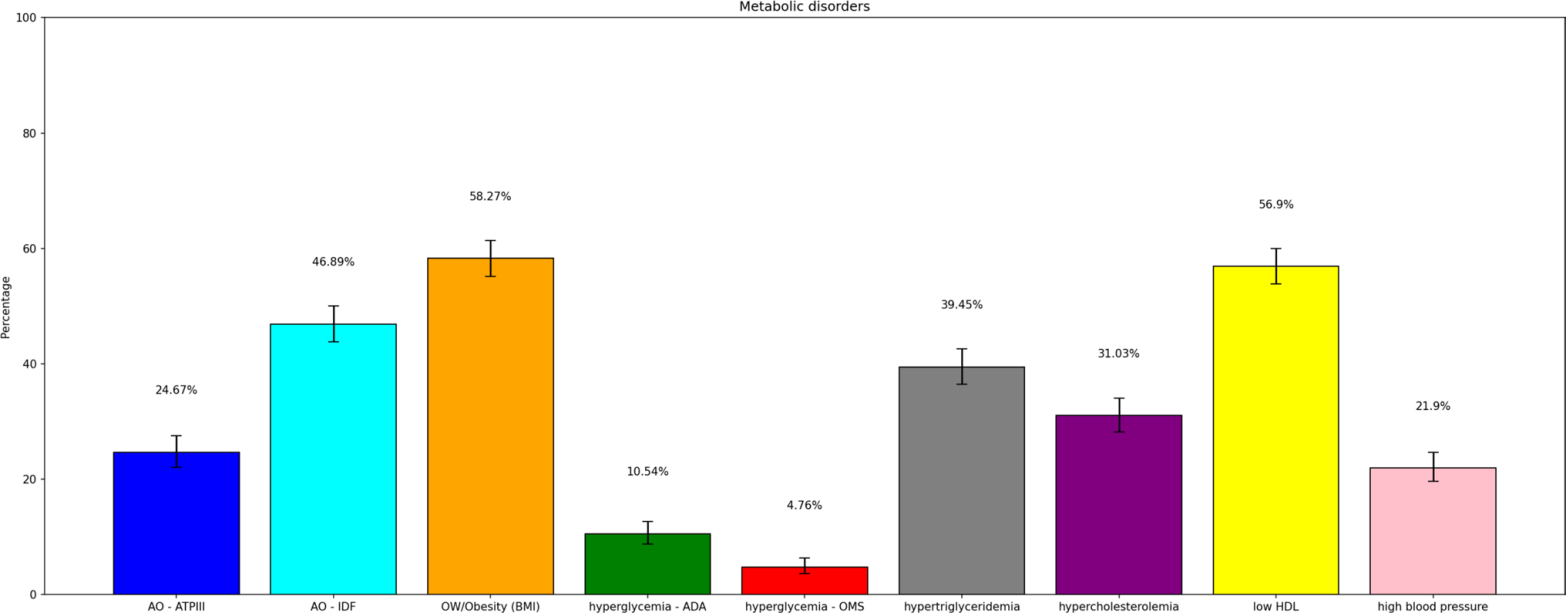
Prevalence of each component of the metabolic state – PERU MIGRANT

Analysis of PERU MIGRANT and VIANEV data revealed consistent patterns in the prevalence of metabolic disorders despite differences in the studied populations. Both studies showed a high prevalence of metabolic alterations, varying according to the diagnostic criteria. You can visualize this in detail in Figs. 3 and 4.

Abdominal obesity presented a high prevalence, with significant differences according to the criteria employed. The IDF criteria resulted in consistently higher prevalences than the ATPIII (64.24% vs 40.55% in PERU MIGRANT, 46.89% vs 24.97% in VIANEV). The prevalence of obesity/overweight by BMI was also high in both studies (64.92% in PERU MIGRANT, 58.27% in VIANEV).

Hyperglycemia showed substantial variation depending on the criterion used. The ADA criteria resulted in notably higher prevalences than the WHO in both studies (64.14% vs 38.44% in PERU MIGRANT, 10.54% vs 4.76% in VIANEV). Among dyslipidemias, low HDL was the most prevalent disorder (76.42% in PERU MIGRANT, 56.4% in VIANEV). Hypertension, although less prevalent, affected approximately one-fifth of both populations.

### Sex differences in the prevalence of metabolic disorders

Table 2 provides a detailed view of the distribution of metabolic alterations by sex. Generally, a trend is observed where women have a higher proportion of multiple metabolic alterations than men, especially in the categories of four or more alterations. In the PERU MIGRANT study, this trend is more pronounced, with women consistently representing a higher percentage in the categories of five, six, and seven alterations across all scenarios. In VIANEV, the trend is similar but even more marked. Women represent significantly more in six and seven alterations across all scenarios.

**Table 2:** Distribution of metabolic alterations by sex in the PERU MIGRANT/VIANEV studies

Metabolic alterations	PERU MIGRANT		VIANEV	
	Sex			
	Female	Male	Female	Male
	n (%)	n (%)	n (%)	n (%)
OA-ATP + Hyperglycemia-ADA				
None	12 (52.17)	11 (47.83)	38 (30.16)	88 (69.84)
One	42 (37.50)	70 (62.50)	102 (48.34)	109 (51.66)
Two	80 (49.69)	81 (50.31)	101 (51.53)	95 (48.47)
Three	74 (48.68)	78 (51.32)	98 (52.97)	87 (47.03)
Four	124 (65.26)	66 (34.74)	85 (62.96)	50 (37.04)
Five	87 (66.92)	43 (33.08)	57 (69.51)	25 (30.49)
Six	54 (71.05)	22 (28.95)	32 (86.49)	5 (13.51)
Seven	11 (57.89)	8 (42.11)	6 (75.00)	2 (25.00)
OA-IDF + Hyperglycemia -ADA				
None	9 (45.00)	11 (55.00)	35 (28.46)	88 (71.54)
One	33 (32.35)	69 (67.65)	80 (44.20)	101 (55.80)
Two	46 (40.71)	67 (59.29)	79 (47.59)	87 (52.41)
Three	88 (56.41)	68 (43.59)	108 (58.38)	77 (41.62)
Four	138 (65.71)	72 (34.29)	101 (64.74)	55 (35.26)
Five	101 (69.66)	44 (30.34)	73 (66.36)	37 (33.64)
Six	56 (62.22)	34 (37.78)	37 (72.55)	14 (27.45)
Seven	13 (48.15)	14 (51.85)	6 (75.00)	2 (25.00)
OA-ATP + Hyperglycemia -OMS				
None	17 (40.48)	25 (59.52)	38 (29.92)	89 (70.08)
One	57 (43.18)	75 (56.82)	103 (48.36)	110 (51.64)
Two	85 (50.00)	85 (50.00)	104 (52.00)	96 (48.00)
Three	91 (56.52)	70 (43.48)	110 (55.84)	87 (44.16)
Four	104 (65.00)	56 (35.00)	78 (59.54)	53 (40.46)
Five	84 (66.67)	42 (33.33)	55 (72.37)	21 (27.63)
Six	38 (64.41)	21 (35.59)	28 (90.32)	3 (9.68)
Seven	8 (61.54)	5 (38.46)	3 (60.00)	2 (40.00)
OA-IDF + Hyperglycemia -OMS				
None	12 (32.43)	25 (67.57)	35 (28.23)	89 (71.77)
One	40 (36.36)	70 (63.64)	81 (44.26)	102 (55.74)
Two	60 (45.45)	72 (54.55)	81 (48.21)	87 (51.79)
Three	113 (62.09)	69 (37.91)	120 (61.22)	76 (38.78)
Four	116 (67.44)	56 (32.56)	96 (61.15)	61 (38.85)
Five	92 (68.15)	43 (31.85)	71 (67.62)	34 (32.38)
Six	43 (55.84)	34 (44.16)	32 (76.19)	10 (23.81)
Seven	8 (44.16)	10 (55.84)	3 (60.00)	2 (40.00)

Notably, men tend to have a more excellent representation in the categories of none or one alteration, especially in the VIANEV study. Moreover, the distribution appears to be more balanced in both studies’ intermediate categories (two and three alterations). Finally, the differences in distribution between the different criteria (ATP vs. IDF for AO and ADA vs. WHO for hyperglycemia) are evident, but the general trend of higher prevalence of multiple alterations in women is maintained.

Table 3 presents the results from ordinal logistic regression analyses examining sex-based disparities in metabolic alterations. The OR of 0.44 (95% CI: 0.35–0.55) for males in the PERU MIGRANT study (using OA-ATP + Hyperglycemia-ADA criteria) indicates that men had 56% lower odds of having a higher number of metabolic alterations compared to women. Similar patterns were observed across all criteria combinations and in both study populations, consistently showing that women had significantly higher odds of presenting multiple metabolic alterations compared to men. For instance, in the VIANEV study, men had 53% lower odds (OR: 0.47; 95% CI: 0.37–0.61) of having a higher number of metabolic alterations than women when using the same criteria.

**Table 3:** Ordinal logistic regression for metabolic state disparities according to sex

Sexo	Metabolic Status							
	OA-ATP + Hyperglicemia-ADA		OA-IDF + Hyperglicemia-ADA		OA-ATP + Hyperglicemia-OMS		OA-IDF + Hyperglicemia-OMS	
	OR	95% IC	OR	95% IC	OR	95% IC	OR	95% IC
PERU MIGRANT								
Female	Ref		Ref		Ref		Ref	
Male	0.44	0.35–0.55	0.40	0.32–0.51	0.45	0.35–0.56	0.41	0.32–0.51
VIANEV								
Female	Ref		Ref		Ref		Ref	
Male	0.47	0.37–0.61	0.48	0.37–0.61	0.53	0.42–0.68	0.51	0.40–0.65

*OR* Odds ratio, *CI 95%* Confidence interval at 95%

## Discussion

### Prevalence of metabolic alterations

When analyzing metabolic alterations in the population, it is crucial to highlight that, regardless of the criteria used, a small proportion of individuals do not present any alterations. Despite some differences in absolute prevalences, the general distribution pattern of metabolic disorders remained similar across studies. Moreover, while classifications such as altered metabolic state (two or more alterations) or metabolic syndrome (three or more alterations) have been established, it is essential to recognize that each of these metabolic markers carries an independent risk of cardiovascular morbidity and mortality [2].

This reality raises two fundamental questions: Are we genuinely facing a metabolic epidemic? Is there a problem with the cut-off points used to define these alterations? Particularly concerning is that, even in the most optimistic scenario, using less stringent cut-off points, the prevalence of metabolic alterations (at least one alteration) reaches 87.04% of the studied population. In the worst-case scenario, this figure rises to 87.55%.

The high prevalence of metabolic alterations in our population (87.04%− 87.55%) is considerably higher than those reported in many international studies. In our research, we found a total prevalence of subjects with metabolically unhealthy state (MUS) of 73.11%. This high figure aligns with the findings of Benziger et al. [14], which concluded that the total percentage of people with MUS was 75.30%. Since the latter was a semi-representative investigation that coincides with the values found in our nationally representative study, there is a strong probability that the number of people with metabolic abnormalities is indeed high in Peru.

At the global level, studies conducted in China, such as Chen et al. [12], reported a prevalence of 42.16%, although it should be noted that they used metabolic syndrome criteria per se to define MUS; and the work of Zhang Y et al. [13] found a prevalence of 52.11%. In Sweden, a study in people over 50 years determined that the prevalence of MUS, through metabolic syndrome criteria, was 14.50%. In the United States, the prevalence was 46% [15]. These differences could be attributed to a combination of genetic factors, unhealthy diet and lifestyle, lower levels of physical activity, socioeconomic inequalities, and cultural differences regarding attitudes toward health and nutrition.

These data suggest that, regardless of how metabolic alterations are defined, most populations present at least one metabolism-related cardiovascular risk factor. This situation poses significant challenges for public health and clinical practice, implying that almost 9 out of 10 individuals might require medical intervention or follow-up to prevent future complications [3, 16, 17].

These findings could have significant implications for health policies, prevention strategies, and the allocation of healthcare resources. Furthermore, they underscore the need to investigate further the factors contributing to this high prevalence of metabolic alterations in the population. Recent studies have shown that factors such as diet, sedentary lifestyle, and chronic stress play a crucial role in developing these alterations [18].

This study’s high prevalence of metabolic alterations aligns with global trends. For example, a recent meta-analysis estimated that the worldwide prevalence of metabolic syndrome is approximately 25%, with significant variations between regions and age groups [18]. However, our findings suggest that, when considering individual alterations, the proportion of the affected population could be much higher.

### Healthy behaviors in Peru

It is essential to consider that healthy behaviors in Peru are significantly diminished, as various studies have demonstrated. Harmful habits such as smoking and alcohol consumption remain at concerning levels or experience marginal reductions. A recent study revealed that the prevalence of smoking in Peruvian adults is 12.2%, while risky alcohol consumption reaches 21.1% [19].

Particularly alarming is the low consumption of fruits and vegetables in the country. Research has shown that less than 10% of the Peruvian population meets the World Health Organization’s recommendation to consume at least 400 g of fruits and vegetables daily [20]. This deficit in the intake of foods rich in essential nutrients and fiber can significantly contribute to metabolic alterations.

While there is debate about the exact prevalence of metabolic alterations in Peru, the existence of an environmental component that justifies the high observed rates cannot be denied. Factors such as rapid urbanization, changes in dietary patterns towards ultra-processed foods, and increased sedentary behavior have been identified as key contributors to the epidemic of metabolic alterations in developing countries like Peru [21]. Furthermore, recent studies have highlighted the influence of social determinants of health on the prevalence of metabolic alterations in Peru. For example, a significant association has been found between socioeconomic status and the prevalence of metabolic syndrome, being higher in lower socioeconomic groups [22].

In this context, it is crucial to implement comprehensive public health strategies that address individual risk factors and social and environmental determinants of health. These strategies should include policies to promote healthy eating, increase physical activity, and reduce tobacco and alcohol consumption, adapted to the Peruvian cultural and socioeconomic context [23].

### Discrepancies in obesity criteria

The ATP III criteria recommend higher cut-off points for AO, resulting in a lower prevalence. In contrast, the IDF criteria, which suggest more specific cut-off points for the Latin population, elevate the prevalence to almost 65% of the Peruvian population [22]. This contrast raises crucial questions: Do 65% of Peruvians have obesity? Or should we seek more specific cut-off points for our population?

When examining nutritional status according to BMI, we find that approximately 60% of the Peruvian population is overweight or obese. However, it is essential to note that the prevalence of obesity by BMI is around 26% [24]. This distinction is crucial, as the psychological impact and clinical implications of labeling a patient as “overweight” differ significantly from diagnosing them with ”obesity”.

Our results clearly reflect these discrepancies, with significant differences in the prevalence of abdominal obesity according to the criteria used. The prevalence according to IDF criteria was approximately 24% higher than with ATP III in both studies, which has important implications for the identification and management of patients at risk. This highlights the need to establish locally appropriate cut-off points for the Peruvian population that better reflect our ethnic, genetic, and environmental characteristics. Developing these population-specific criteria would improve the accuracy of metabolic risk assessment, prevent both over-diagnosis and under-diagnosis, and allow for more effective targeting of preventive interventions to those who would truly benefit from them.

Recent research suggests the need to reevaluate cut-off points for AO in specific populations. A study by Vasquez-Romero proposes that the optimal cut-off points for WC in the Peruvian population could be different from those currently used, which could significantly impact the reported prevalence of AO and metabolic syndrome [24]. Thus, the choice of diagnostic criteria affects epidemiological statistics and has important implications for clinical practice and public health policies.

It is essential to highlight that, regardless of its combination with hyperglycemia, AO, according to IDF criteria, appears to be the main factor responsible for the high prevalence of metabolic alterations. This underscores the need to critically evaluate diagnostic criteria and their applicability in different populations [25].

Therefore, it is fundamental to reconsider and possibly reclassify the cut-off points used to define AO in Peru, seeking more local and specific criteria. This would improve the accuracy of our epidemiological assessments and allow for more targeted and effective public health interventions.

### Discrepancies in hyperglycemia

There are also significant discrepancies in diagnostic criteria for hyperglycemia. For years, the ADA has recommended considering values above 100 mg/dl as the cut-off point to define alterations in glucose metabolism. In comparison, the WHO maintains its position of considering values above 110 mg/dl [8, 9].

This divergence creates essential challenges in diagnosing and managing hyperglycemia in patients. Recent studies have found that cardiovascular risk increases significantly only from values above 110 mg/dl, which supports the WHO’s position [26]. This finding has important implications for both clinical practice and public health.

When comparing the prevalences of hyperglycemia according to WHO and ADA criteria, a considerable reduction is observed when using the higher WHO cut-off point. This can be explained by the concentration of a significant number of patients between 100 and 110 mg/dl. This difference is not trivial, as labeling a patient with hyperglycemia not only increases their concerns but can also lead to the application of medical treatments based on medications, which are not exempt from adverse effects.

A systematic review by Richter B et al. found that individuals with fasting glucose between 100–109 mg/dl had a lower risk of progression to type 2 diabetes mellitus than those with levels between 110–125 mg/dl [27]. Furthermore, a meta-analysis by Huang et al. [28] suggests that optimal cut-off points for prediabetes diagnosis may vary according to ethnicity and geographic region. This highlights the importance of considering population-specific factors when establishing diagnostic criteria.

Thus, it is crucial to consider these implications when diagnosing and treating hyperglycemia. While monitoring glucose levels in all patients is essential, the therapeutic approach may vary. For patients with values between 100–110 mg/dl, interventions could focus more on lifestyle changes, while for those above 110 mg/dl, more aggressive pharmacological interventions could be considered.

Therefore, it is essential to re-evaluate the diagnostic criteria for hyperglycemia, considering not only numerical cut-off points but also the clinical impact and implications for treatment. More specific research on Latin American populations is needed to determine the most appropriate cut-off points and effective management strategies.

### Sex disparities

Sex disparities in metabolic alterations are a topic of growing interest in epidemiological and clinical research. Our study reveals a consistent trend: Women present a higher prevalence of multiple metabolic alterations than men, regardless of the criteria used to define these alterations.

The results of our ordinal logistic regression consistently confirm this trend, showing that men have between 47–60% lower probability of presenting metabolic alterations compared to women (OR between 0.40–0.53), regardless of the diagnostic criteria used. This association was robust across both study populations, despite the temporal and methodological differences between the PERU MIGRANT and VIANEV studies.

Our bivariate analysis shows this trend: women are overrepresented in categories with four or more metabolic alterations, while men tend to have greater representation in the categories of none or one alteration. These findings are consistent with recent studies that have found that women tend to present more metabolic alterations than men [5].

Our findings regarding sex-based differences in metabolic alterations align with previous Peruvian studies [29–31], which similarly found women more susceptible to these conditions, though this contrasts with some international research that often identifies males as having higher risk [32–34]. This finding is also consistent with previous studies in Latin America. For example, Márquez-Sandoval et al. [35] found a higher prevalence of metabolic syndrome in women in several Latin American countries.

Moreover, hormonal differences and body composition between men and women could influence metabolic risk. A study by Mauvais-Jarvis and Hevener [36] highlights how estrogens affect glucose and lipid metabolism differently in women. The greater metabolic vulnerability among women likely stems from multiple factors. Body fat distribution plays a crucial role—premenopausal women tend to develop peripheral fat patterns with subcutaneous accumulation, while men and postmenopausal women more commonly exhibit central or android obesity, the latter being more strongly associated with cardiovascular disease risk. Furthermore, research indicates that inflammatory processes contribute to increased cardiovascular risk, with potentially stronger effects in women [37, 38].

On the other hand, differences in gender roles, access to healthcare, and physical activity patterns could contribute to these disparities. A study by Creber in Peru found that women had lower physical activity than men, which could increase their metabolic risk [39]. These sex disparities we observed may be partially explained by behavioral patterns in Peru, where women typically have more limited opportunities for physical activity and follow dietary practices shaped by traditional gender roles.

This combination of biological differences and socially-determined lifestyle factors helps explain the consistent sex-based disparities evident across both study populations we analyzed. Our findings and existing evidence underscore the need for a sex-differentiated approach to evaluating and managing metabolic alterations. More research is needed, particularly in Latin American populations, to better understand the underlying reasons for these disparities and to develop prevention and treatment strategies considering these differences between sexes.

### Importance for public health

This study is of fundamental importance for public health in Peru and internationally. By revealing the high prevalence of metabolic alterations in the Peruvian population, it provides a solid basis for formulating more effective and targeted public health policies. Identifying discrepancies in diagnostic criteria and their implications for the reported prevalence of abdominal obesity and hyperglycemia underscores the need to re-evaluate and possibly adapt these criteria to the specific characteristics of the Peruvian and Latin American populations in general.

At the international level, this study contributes to the growing body of evidence suggesting the need for population-specific diagnostic criteria. This is particularly relevant in an increasingly globalized world, where health recommendations are often applied universally without considering ethnic and regional variations. The findings of this study can influence how international guidelines for diagnosing and managing metabolic alterations are developed and used, promoting a more personalized and culturally sensitive approach in preventive medicine and global public health.

The sex disparities identified in our study underscore the need to develop differentiated interventions. Peruvian women could particularly benefit from programs that address the specific barriers they face in adopting and maintaining healthy lifestyles, including considerations regarding domestic roles, access to safe spaces for physical activity, and culturally appropriate nutritional education.

### Conclusions and recommendations

This study reveals a high prevalence of metabolic alterations in the Peruvian population, with significant variations according to the diagnostic criteria. The observed discrepancies in the prevalence of AO and hyperglycemia, depending on the cut-off points employed, underscore the need to re-evaluate these criteria for the Peruvian population and possibly for other Latin American populations. Furthermore, the observed differences between sexes in the prevalence of metabolic alterations, particularly in abdominal obesity, suggest the need to consider sex-differentiated approaches in prevention and management strategies.

The findings of this study have important implications for clinical practice and public health policies. Even with more conservative criteria, the high prevalence of metabolic alterations indicates an urgent need for preventive interventions at the population level. However, the variability in diagnoses according to the requirements also suggests the importance of a more nuanced and personalized approach in assessing and managing individual metabolic risks.

Additional studies are recommended to determine the optimal cut-off points for abdominal obesity and hyperglycemia in the Peruvian population, considering ethnic, regional, and sex differences. Meanwhile, at the public health policy level, it is recommended to implement comprehensive strategies that address metabolic risk factors from multiple angles. This includes public education campaigns on healthy lifestyles, policies that promote balanced nutrition and physical activity, and improvements in access to preventive health services. Additionally, it is suggested that national clinical guidelines be developed that consider this study’s findings, guiding health professionals on how to interpret and manage metabolic alterations in the specific context of the Peruvian population.

## Data Availability

The data supporting the findings of this study can be accessed by the original research paper at the following link: • PERU MIGRANT https://figshare.com/articles/dataset/PERU_MIGRANT_Study_Baseline_dataset/3125005. •VIANEV: https://www.datosabiertos.gob.pe/dataset/estado-nutricional-en-adultos-de-18-59-a%C3%B1os-per%C3%BA-2017-%E2%80%93-2018
